# Surgical Capacity in Rural Southeast Nigeria: Barriers and New Opportunities

**DOI:** 10.5334/aogh.3367

**Published:** 2021-11-25

**Authors:** Aloysius U. Ogbuanya, Stanley Nnamdi C. Anyanwu, Akuma Ajah, Onyeyirichi Otuu, Nonyelum Benedett Ugwu, Emmanuel A. Boladuro, Williams Otu Nandi

**Affiliations:** 1 Alex Ekwueme Federal University Teaching Hospital, Abakaliki (AEFUTHA), Ebonyi State, Nigeria; 2 Ebonyi State University, Abakaliki (EBSU), Ebonyi State, Nigeria; 3 Nnamdi Azikiwe University Teaching Hospital, Nnewi (NAUTH), Anambra State, Nigeria; 4 Nnamdi Azikiwe University, Awka, Anambra State, Nigeria; 5 AEFUTHA Lecturer, EBSU, Nigeria; 6 Enugu State Ministry of Health, Enugu, Nigeria

## Abstract

**Background:**

Remarkable gains have been made in global health with respect to provision of essential and emergency surgical and anesthesia care. At the same time, little has been written about the state of surgical care, or the potential strategies for scale-up of surgical services in sub-Saharan Africa, southeast Nigeria inclusive.

**Objective:**

The aim was to document the state of surgical care at district hospitals in southeast Nigeria.

**Methods:**

We surveyed 13 district hospitals using the World Health Organization (WHO) tool for situational analysis developed by the “Lancet Commission on Global Surgery” initiative to assess surgical care in rural Southeast Nigeria. A systematic literature review of scientific literatures and policy documents was performed. Extraction was performed for all articles relating to the five National Surgical, Obstetric and Anesthesia Plans (NSOAPs) domains: infrastructure, service delivery, workforce, information management and financing.

**Findings:**

Of the 13 facilities investigated, there were six private, four mission and three public hospitals. Though all the facilities were connected to the national power grid, all equally suffered electricity interruption ranging from 10–22 hours daily. Only 15.4% and 38.5% of the 13 hospitals had running water and blood bank services, respectively. Only two general surgeon and two orthopedic surgeons covered all the facilities. Though most of the general surgical procedures were performed in private and mission hospitals, the majority of the public hospitals had limited ability to do the same. Orthopedic procedures were practically non-existent in public hospitals. None of the facilities offered inhalational anesthetic technique. There was no designated record unit in 53.8% of facilities and 69.2% had no trained health record officer.

**Conclusion:**

Important deficits were observed in infrastructure, service delivery, workforce and information management. There were indirect indices of gross inadequacies in financing as well.

## Introduction

Despite growing need, the development and delivery of surgical and anesthesia care in low and middle-income countries (LMICs) has been nearly absent from the global health discourse [1,2,3,4]. Studies have shown that the rate of major complications caused by surgery for hospitalized patients in developed countries is 3–6%, accounting for 0.4–0.8% of the mortality in those countries, as opposed to the mortality rate of 5–10% in developing countries [1]. Current published data have estimated that 313 million operations are performed annually, with most taking place in high-income and upper-middle income countries [1,4]. Unfortunately, only 6% of the 313 million procedures undertaken worldwide each year occur in the poorest countries where over a third of the world’s population lives [1,4,5,6,7].

Despite steady gains in raising the profile of surgery in the public health domain, untreated surgical conditions continue to plague certain societies, causing 25% of the avoidable mortalities and up to 40% of avoidable morbidities in LMICs [1,7]. Indeed, an additional 143 million surgical procedures are required each year to meet the need in LMICs [1,7]. A review of National Health Strategic Plans in 43 sub-Saharan African countries revealed a lack of surgical prioritization [7]. In that survey, 19% of the participant nations had no mention of surgery, and only 47% referenced traumatic conditions despite high injury mortality rates in those regions [7].

The overall disease burden associated with surgical conditions in sub-Saharan Africa is estimated at 38 DALYs (disability adjusted life years) lost per 1000 population [8,9]. This estimate is higher than values quoted in other regions of the world and is mainly due to injuries (15/1,000), obstetric complications (6/1,000), malignancies (3/1,000), perinatal conditions (3/1,000), congenital anomalies (3/1,000), and cataract and glaucoma (2/1,000) [8,10]. The estimated cost of surgical DALY gained at a district hospital is in the range of USD19-102 [3].

Briefly, public health programs like basic immunization programs, malaria prevention and treatment, oral rehydration therapy for diarrhea diseases, and anti-retroviral therapy for HIV infection in sub-Saharan Africa are estimated to cost USD 10-1,494/DALY averted [8]. Despite the lower cost per surgical DALY averted compared to most of these programs, surgical diseases are still largely neglected by the global health community when supporting interventions that are already in the public health domain in sub-Saharan Africa [8,10,11,12]. Interestingly, surgery was elevated to a global health priority in 2015 by the adoption of the World Health Assembly resolution that called for the strengthening of emergency and essential surgical and anesthesia care as an integral component of universal health coverage (UHC) [1,13].

In addition, the Lancet Commission on Global Surgery (LCOGS) was launched in January 2014 using international, multidisciplinary team of 25 commissioners, supported by advisors and collaborators in more than 110 countries and six continents, and it has since proved to be a useful instrument for global surgery advocacy [1,14]. In pursuit of its primary objective aimed at bridging the gap between different sectors of health managers and creating smooth, functional health care coordination, LCOGS recommended the development of National Surgical, Obstetric and Anesthesia Plans (NSOAPs) to form and sustain improvement on five major domains: service delivery, training and workforce, economics and finance, infrastructure, and information management [1,14,15]. The framework of strategic areas developed in each of the major domains formed the backbone for analysis to identify the strengths and weaknesses of a health system so that an informed decision on the areas of priority can be made [1,14,16,17].

In LMICS, especially at district centres in sub-Saharan African communities, surgical capacity and safety leaves a lot to be desired, but no formal study has been done to analyse the current scope, weaknesses and needs of the underserved system. The aim of this study was to document the current state of the art on the five main domains of surgical systems strengthening at district hospitals in southeast Nigeria. Hopefully, data derived from this study will inform decision on areas of advocacy for development of each domain.

## Materials and Methods

### Setting and Deign

The study involved 13 hospitals located in rural and semi-urban areas of two states (Enugu and Ebonyi) of Southeast geopolitical zone of Nigeria. The Enugu and Ebonyi States have an estimated land mass of 7,161 km^2^ and 5,533 km^2^, respectively [18]. According to National population commission of Nigeria, the projected population of the Enugu and Ebonyi States in 2016 is 4,411,100 and 2,880,400, respectively [18]. The current survey was conducted from January 2018 to December 2018 (12 months) and reported on the activities of the district hospitals between January 2014 to December 2018. Rural Southeast Nigeria comprised predominantly agrarian communities followed by traders and artisans, in that order. The eligibility criteria for the district centres included location in rural or semi-urban areas, availability of at least 10 bed spaces, capacity to perform surgical operations and an informed consent to participate in the study.

### Procedure

The WHO tool for situational analysis to access surgical and anesthesia care contained in the Lancet Commission on Global Surgery (LCoGS) [1] was used to collate data for this study. In 2015, the Lancet Commission on Global Surgery report recommended the development of National Surgical, Obstetric and Anesthesia Plans (NSOAPs) to serve as a roadmap to improvement across five main domains [1]. The NSOAPs guidelines formed the basis of the WHO situational analysis tool used in this survey. The tool was distributed to the health managers of the 13 selected district hospitals. The facilities included one health centre, two general hospitals, four mission hospitals and six private health facilities. The tool comprised questions that were grouped into sections according to the five main domains of NSOAPs [1].

Section 1 contained questions that concerned infrastructure and proportion of hospitals fulfilling safe surgery criteria. Questions on workforce and service delivery were contained in sections II and III, respectively. Furthermore, section IV contained relevant items on financing and economics of the hospitals, while section V was designed to extract data relating to information management.

In addition to the questionnaires, interviews with health facility personnel were arranged to generate more robust data. These data were subsequently entered into a proforma.

### Data Analysis

Data was analyzed using Statistical Package for Social Sciences (SPSS) version 22.0 (IBM, Chicago, IL, USA,2015). Data were presented in tabular and descriptive forms. Descriptive statistics were used to compare the variables for the different hospitals. Confidence interval was calculated at 95% level and significance at 5% probability level (P < 0.05).

#### Ethical Approval

The protocol for this study was approved by the “Ethical Research Board” of Bishop Shanahan Specialist Hospital, Nsukka, Enugu state, Nigeria. The approval number was **BSSH/ERB/17/52**. Ethical approval was waived by the remaining 12 health facilities since human subjects were not directly involved.

## Results

### Characteristics of the health institutions:

All 13 hospitals selected were capable of delivering both elective and emergency surgical services. Seven (53.8%) hospitals were located in semi-urban settlements, the rest (46.2%) were found in the rural areas. The road networks to all the hospitals in the rural settlement were in a deplorable state.

### 1 Infrastructure

The infrastructural facilities available in the selected hospitals are represented below (***Table 1***). The percentage availability of the various infrastructures in the government-owned (public), mission and private hospitals were equally compared. All the selected hospitals have a designated operating room. However, none had a modern design—floor to ceiling tilling, cul-de-sac positioning, adequate ventilation systems, and permanently closed windows. None of the facilities had more than one functional operating room. There was a total of 615 beds; 460 (74.8%) belong to mission hospitals, 85(13.8%) to private and 70(11.4%) to public hospitals. None of the facilities had an intensive care unit, computed tomography scan, defibrillator, electrocardiographic (ECG) unit or dialysis section.

**Table 1:** Percentage availability of infrastructure in the facilities (%).


INFRASTRUCTURE	PRIVATE (N = 6)	MISSION (N = 4)	PUBLIC (N = 3)	TOTAL (N = 13)	P-VALUE

Operating Room	100.0	100.0	66.7	100.0	1.000

Running Water	16.7	50.0	0.0	15.4	0.650

^a^Electricity	100.0	100.0	100.0	100.0	1.000

Blood Bank Services	0.0	75.0	66.7	38.5	0.790

Accident & Emergency	0.0	75.0	33.3	30.8	0.410

Oxygen Supply	33.3	100.0	33.3	53.8	0.042

Laboratory services	66.7	100.0	100.0	84.6	0.782

^b^X-ray Unit	16.7	75.0	0.0	30.8	0.021

Post-Op care Unit	16.7	25.0	0.0	15.4	0.133

^b^Anesthetic Machine	0.0	75.0	0.0	23.1	0.001

Power Generator	83.3	100.0	66.7	84.6	0.972

^b^Medical record Unit	16.7	75.0	66.7	53.8	0.644

Pharmacy Unit	33.3	100.0	66.7	61.5	0.047

Electrosurgical Set	16.7	50.0	0.0	23.1	0.038

^c^Endoscopic Unit	16.7	0.0	0.0	7.7	0.510

Autoclave	66.7	100.0	66.7	76.9	0.800

Suctioning Machine	16.7	100.0	33.3	46.2	0.030

^b^Theatre Light	16.7	75.0	0.0	30.8	0.024

^b^Operating Table	33.3	100.0	66.7	61.5	0.631

Ultrasound Unit	33.3	75.0	66.7	53.8	0.771

Incinerator	50.0	75.0	66.7	61.5	0.802


Op = operative; a = interrupted supply; b = functional; c = available but not functional.

An endoscopic facility (upper and lower GI endoscopes) is available but not functional in one of the mission hospitals. Broadly, three (23.1%) were primary hospitals while the rest (10, 76.9%) were secondary hospitals. Though electricity from the national grid was present in all the facilities, it was habitually interrupted for at least 10 hours daily. There was a statistically significant difference between services in mission and other health facilities in terms of availability of a functional anesthetic machine (p = 0.001), electrosurgical unit (p = 0.040) and suctioning machine (p = 0.030). The toilet facility was water cistern in 10 (76.9%), but pit method in 3 (23.1%) centres.

#### 1.1 Essential supplies

Each of the essential surgical supplies was found to be lacking in at least one or more private or public health facilities during the time of survey. However, only nasogastric tubes, nasopharyngeal airways, resuscitation bags and eye protection were found to be absent in at least one or more mission hospitals (***Table 2***).

**Table 2:** Availability of Essential supplies.


SUPPLIES	PERCENTAGE AVAILABILITY (%)			P-VALUE

	PRIVATE	MISSION	PUBLIC	

Examination gloves	66.7	100.0	66.7	0.022

Sterile (surgical) gloves	66.7	100.0	66.7	

Nasogastric tubes	33.3	75.0	0.0	

Nasopharyngeal Airway	33.3	75.0	33.3	

Resuscitator bag	0.0	50.0	33.3	

Intravenous infusion set	83.3	100.0	66.7	

Nose mask	50.0	100.0	33.3	

Sterile cap	66.7	100.0	33.3	

Urethral catheter	50.0	100.0	33.3	

Eye protection	0.0	25.0	0.0	

Hand washing soap	33.3	100.0	33.3	

Intravenous canula	83.3	100.0	66.7	

Urine bag	66.7	100.0	66.7	


### 2 Workforce/Personnel

There were two general surgeons, two orthopedic surgeons, three urologists, 10 obstetricians/gynecologists and four specialist anesthesiologists (one board certified and 3 senior residents). None except one obstetrician were permanent staff. Only one mission hospital contracted the services of a neurosurgeon, who also provided the instruments for surgical procedures. There was a total of 225 personnel in the surgical/anesthetic section of the 13 institutions. Of these, 29 (12.9%) were general practitioners performing surgery, 8 (3.6%) were board certified surgeons, 10 (4.4%) were obstetricians/gynecologists, 4 (1.8%) were specialist anesthesiologists, 13 (5.8%) were nurse anesthetists, 10 (4.4%) were trainee surgeons and obstetricians, 38 (16.9%) were midwives and perioperative nurses, and the remaining 113 (50.2%) were support staff in accident and emergency, theatre, surgical wards and clinics. The details of the distribution of the personnel are shown below (***Table 3***).

**Table 3:** Workforce for the District centres.


PERSONNEL	PRIVATE	MISSION	PUBLIC	TOTAL

General surgeon	1	2*	1	2

Orthopedic surgeon	0	2	0	2

Gynecologist/Obstetrician	2*	8	1	10

Urologist	1	2	0	3

Neurosurgeon	0	1	0	1

Anesthesiologist	1	2*	0	2

Other doctors performing surg.	7	13	10	30

Nurse anesthetist	3*	11	2	13

Non-physician/Nurse anesthetist	8	2	0	10

Surgical Technician	0	3	0	3

Orthotist	0	1	0	1

Perioperative nurses	0	3	1	4

Physiotherapist	1	2	0	3

Radiographers	2*	5	1*	5

Midwives	3*	23	9	34

Other paramedics	32	41	16	89

Trainee surgeons on rural posting	0	4	0	4

Trainee Gynecologist on posting	0	6	0	6


* Covers more than one health facility.

### 3 Service delivery

#### 3.1 Surgical procedures

All the health facilities were able to perform minor surgical procedures like incision and drainage, wound debridement, suturing of lacerations and superficial lump excisions. Intermediate procedures like herniorrhaphy, appendectomy, excision biopsies were also done in all the facilities (***Table 4***). The average annual surgical output was 360 for each mission hospital, 134 for each private hospital and 71 for each public hospital.

**Table 4:** Surgical/Anesthetic procedures performed (service delivery).


PROCEDURE	PERCENTAGE AVAILABILITY (%)				

	TOTAL	PRIVATE	MISSION	PUBLIC	P-VALUE

**General/Urological surgery**:					

Appendectomy	100.0	100.0	100.0	100.0	1.00

Herniorrhaphy	100.0	100.0	100.0	100.0	1.00

Laparotomy	84.6	100.0	100.0	33.3	0.033

Thyroidectomy	23.1	16.7	50.0	0.0	0.045

Mastectomy	46.2	50.0	75.0	0.0	0.074

Core Needle (Tru-cut) Biopsy	53.8	50.0	100.0	0.0	0.021

Chest Tube Insertion	30.8	16.7	75.0	0.0	0.022

Prostatectomy	30.8	33.3	50.0	0.0	0.062

**Orthopedic/Plastic surgery**:					

Limb Amputation	30.8	16.7	75.0	0.0	0.010

Close reduction and POP	53.8	66.7	100.0	0.0	0.076

Open reduction and Internal Fix	30.8	16.7	50.0	0.0	0.084

Skin grafting	38.5	33.3	50.0	0.0	0.254

**Obstetric/Gynecology procedure**					

Caesarean section	84.6	83.3	100.0	66.7	0.613

Hysterectomy	61.5	66.7	100.0	0.0	0.048

Myomectomy	84.6	100.0	100.0	33.3	0.026

**Anaesthesia**					

General	53.8	66.7	75.0	0.0	0.031

Regional	61.5	66.7	100.0	33.3	0.077


POP = plaster of Paris; fix= fixation.

#### 3.2 General surgical and trauma procedures

All the facilities except the health centre had the capacity to carry out laparotomy (92.3%) and the majority performed foreign body extraction (84.6%), hemorrhoidectomy (61.5%) and male circumcision (69.2%). Chest tube insertion, thyroidectomy, mastectomy and tracheostomy were reported in less than half of the facilities. In seven institutions (53.8%), trucut biopsy and fistulectomy were routinely performed. Only four (30.8%) centres performed major amputation and skin grafting while three (23.1%) reported open reduction with internal fixation and/or external fixation. Caesarean section and myomectomy were reported in 84.6% of the health facilities surveyed. General anesthesia with endotracheal intubation and spinal anesthesia were not reported in any of the public facilities due to absence of physician anesthesiologist and functional anesthetic machine/monitor. All the cases in the public hospitals were performed under sedation using ketamine alone or with diazepam and promethazine or alternately local infiltrative anesthesia. All the health facilities offered hydrocelectomy, but only one private and two mission hospitals offered urethroplasty and prostatectomy regularly.

### 4 Information management

The majority (9,69.2%) of the health institutions do not have a trained health record officer, and lacked systematic case note filing. There was no designated record unit in more than half (7,53.8%) of the institutions and the medical record attendant was improvised for record officer in one of the mission hospitals. Computer system and internet services were available in only five (38.5%) and two (15.4%) health facilities, respectively. Clinical history, operation notes, anesthesia records, partogram and follow up records were incomplete in 12 (92.3%), 13 (100.0%), 13(100.0%), 10(76.9%) and 11 (84.6%) health facilities, respectively. None of the health facilities has a functional surgery-related library. The human and technology resources for information management recorded in this study are shown below (***Table 5***).

**Table 5:** Human and technology resources for information management.


PARAMETERS	PERCENTAGE AVAILABILITY (%)		

	PRIVATE	MISSION	PUBLIC

Health Record Officer	**0.0**	**75.0**	**33.3**

Medical record Unit	16.7	75.0	66.7

Computer system	33.3	75.0	0.0

Internet Services	16.6	50.0	0.0

Medical record Attendant	0.0	25.0	0.0

Systematic Filing of case notes	16.7	50.0	33.3

Functional Library Unit	0.0	0.0	0.0


### 5 Financing

Health financing was difficult to estimate in this study due to poor budgetary planning and poor documentation of expenditures. Only four (2 mission, 1 public and 1 private) hospitals were registered with National Health Insurance Scheme (NIHS); the rest operated ‘pay out-of-pocket’ by patients. It was difficult to estimate the proportion of patients at risk of catastrophic (defined as medical expenditure greater than 40% annual household income) and impoverishing (defined as medical expenditure that leads to forcing a family below the poverty line) expenditures from seeking surgical care in this study due to poor documentation. However, during the period between January to December 2018, 12, 26 and 15 patients were designated ‘awaiting bill settlement’ after discharge in private, mission and public hospitals, respectively. Of the 53 patients reported as ‘awaiting bills’ at the time of this survey, about a third (17, 32.1%) were said to have stayed over one month in the awaiting bill list before offsetting the medical bills. The operational costs for surgery for each health facility could not be computed due to lack of consistency in patients’ billing and expenses (some items were procured by patients while others were provided by the health institutions and the pattern was not regular).

## Discussion

This assessment was undertaken to highlight the surgical capacity at district hospitals in southeast Nigeria in order to identify areas with shortcomings and compare results with data from similar studies in Africa and other developing economies. Data obtained in this study showed significant deficiencies in all five domains assessed, though results from government-owned hospitals were disturbingly inadequate but overlapped with observations made in similar surveys in Nigeria and other African series [2,5,11,12,14,17,19,20,21].

Traditionally, surgery and anesthesia services have often been erroneously assumed to be expensive, technologically demanding and best suited for large hospitals where infrastructural facilities and specialists are found. It is against this backdrop that policy makers and health managers of public health facilities in many LMICs assign limited surgical and anesthesia care to district centres, thereby making safe surgery and anesthesia inaccessible to the vast majority of people that reside in the rural and semi-urban areas of resource-constrained nations [1,2,8,17,20,21]. Interestingly, this survey presents the first snapshot of the surgical capacity in district hospitals in southeast Nigeria and found that, though surgical and anesthesia services were performed in most facilities surveyed, urgent actions are needed to upgrade to the minimum requirements recommended by Lancet Commission on Global surgery [1,2,8,14,17]. Major gaps in the infrastructural, financial, information and human resources needed to deliver safe and adequate surgical and anesthesia care were identified and this study was set to make advocacies that can help to plan interventions by governments, missionaries and private health managers in our setting.

In recent time, surgical and anesthesia care have been recognized as an integral component of a national health system in countries at all levels of development [1,8]. Despite the global clamor for greater access to safe surgical care, the growth and discussions on surgical and anesthesia services in district settings of most LMICs, including southeast Nigeria, have been largely stunted and nearly absent from the global health discourse [1,8,12,20,21]. The synopsis of key findings has been presented in descriptive and tabular forms and the results under the five main domains are discussed in the individual sections below.

## Infrastructure

We observed important deficits in the availabilities of basic infrastructures in the surveyed district hospitals. The first table summarized the percentage availability of the infrastructures with striking gross inadequacies in items like running water, post-operative care units, electrosurgical units, endoscopic facilities, anesthetic machines and x-ray units. Though electricity from a national grid was available in all the facilities, interruptions ranging from 10–24 hours, 1–3 weeks and 1–2 months were quoted by the hospital directors during interview.

In Sierra Leone, the situation was more worrisome [19]. Kingham and colleagues found that five (50.0%) of 10 public hospitals surveyed depended solely on generator power, but owing to scarcity and cost of fuel, patient families were sometimes required to provide the necessary fuel to power generators during surgical procedures [19]. Similarly, in 30.8% (2 public and 2 private) of the hospitals surveyed in this study, patients and relatives depended mostly on private torch light and rechargeable lanterns at night time rather than electricity. Even in the centres with electricity supply, lighting was a problem, given that there were approximately six individual light bulbs that worked in all of the dome lights throughout Sierra Leone [19]. The authors equally reported on acute shortages of beds, oxygen supply, running water and a dearth of anesthetic machines [19]. Reports culled from similar studies in other parts of Nigeria, Tanzania, Gambia, Sudan and a multinational African study conform with the above findings [2,5,17,21,22]. The reasons adduced for the scarce equipment and infrastructural availability in most series were overbearing costs, lack of maintenance or repair, and limited expertise [2,5,17,21,22].

A recent multinational study on equipment for essential surgical care involving nine countries in Africa showed, overall, that the availability of surgical equipment was less in public district than in private hospitals, akin to observations made in this study [5]. A recent review from Tanzania showed that in 2016, only 36% of all blood need in the country was met [14]. Paradoxically, it was equally reported that up to three quarters of cross-matched blood at one of the regional hospitals in Tanzania was unutilized and led to significant wastage within the period under review [14]. The above illustrations perhaps mirror the state of the art in most African nations and point to a major gap in the planning and management of health resources.

## Workforce

Curiously, our observation that only eight surgeons (2 general, 2 orthopedic, 3 urologists and a neurosurgeon) and four physician anesthetists covered the 13 district hospitals, and that none were fulltime staff of the hospitals, conforms with previous findings in Nigeria and other parts of Africa [2,5,14,17,19]. Of the 10 obstetrician/gynecologists, only one was employed on fulltime basis by a mission hospital. The majority (85.2%) of the anesthetic personnel were either nurse anesthetists or non-physician anesthetists. In Gambia, Adams and coworkers observed similar scarce human resources and found that most facilities (83.3%) had several paramedics and midwives who performed minor surgical operations [21]. In three (16.7%) of the 18 facilities studied in Gambia, the hospital workforce relied entirely on paramedical staff to perform basic surgical procedures [21]. Similarly, anesthesia was delivered by anesthesiologists in 22.5% of the centres, general doctors in 5.6% and non-physicians in the remaining 71.9% of the health facilities [21]. The acute shortage of anesthetic workforce probably explains why the prevalent anesthetic technique in the Gambian series was predominantly ketamine-based intravenous anesthesia [21].

In southern Nigeria, Jaymie and colleagues reported on a large series involving 41 health facilities with a total of 117 surgical and anesthesia personnel [2]. Of this staff strength, 52.1% were general practitioners performing surgery, 22.2% were board-certified specialist surgeons, 15.4% were nurse anesthetists and 5.1% were specialist anaesthesiologists [2]. The remaining 5.2% were theatre support staff [2]. Only one anesthesiologist was available in the 16 secondary hospitals [2]. The above illustrates the unmet need for surgical personnel in LMICs, especially in sub-Saharan Africa, where an emerging trend in surgical diseases has been reported with attendant increases in demand for emergency and essential surgical and anesthesia care [1,7,8].

Regrettably, brain drain is a recognized phenomenon in LMICs, Nigeria inclusive. Over the years, particularly in recent time, this has led to specialists in the area of surgery, anesthesia and gynecology leaving in droves to Western countries in search of better jobs and living conditions [23]. The shortage of health personnel, especially for the surgeons and anesthesiologist cadres, hampered the provision of surgical and anesthesia services in the selected facilities in this study. According to reports from some of the surveyed health facilities, non-physician health workers in the neighborhood with little or no formal surgical training occasionally undertake basic surgical procedures to compensate for the lack of trained personnel. Subsequently, many life-threatening complications like enterocutaneous fistula, urinary fistula, exsanguinating haemorrhage, tumor implantation and entrapment of gut during hernia repair were referred from the non-physician health workers to the district hospitals from time to time.

Experience from rural southern Sudan, where local medical practitioners were trained in basic surgical skills, showed that 42% of the procedures they performed were hernia repairs and 77% of their cases were elective [22]. The authors concluded after 6 years that the need for surgical care was still unmet and that 10% to 20% of deaths in operated young adults were due to iatrogenic injuries and directly attributable to inadequate surgical knowledge [22]. In summary, inadequate human resources constituted a significant problem with surgical care in this study, in agreement with previous reports from Tanzania, Gambia, Sierra Leone and Nigeria [2,12,14,17,19,20,21,24].

The realization of this huge unmet need perhaps prompted investigators working in Nigeria to recommend short training programs for physicians at the primary and secondary levels of care and regular revision courses for nurse anesthetists to strengthen the district hospitals in the area of surgical and anesthetic services [17]. The authors further emphasized the need for government incentives for rural health workforce to stimulate urban to rural shift in manpower [17]. Globally, the clamor for Universal Health Coverage (UHC) has reached a crescendo in recent times, leading to the emergence of programs like the Global Initiative for Emergency and Essential Surgical Care (GIEESC), the Bellagio Essential Surgery Group (BESG) and Lancet Commission on Global Surgery (LCOGS) [1,8,14,17]. Indeed, the UHC is a work in progress, hence LCOGs prioritized increased access to surgery and development of National Surgical, Obstetric and Anesthesia Plans (NSOAPs) to serve as roadmaps to improvement across the five main domains: infrastructure, workforce, service delivery, information management and financing [1,8,14].

## Service Delivery

The ability of the various hospitals to provide several basic and major surgical procedures, though commendable, still fell short of the global standard [1,8,13]. The observation in the results section that the average annual surgical output (procedures) was 360 for each mission hospital, 134 for each private hospital and 71 for each public hospital is remarkable. Put differently, the ratio of annual surgical output is 1:2:5 for public, private and mission hospitals, which is a reflection of the surgical volumes in the individual hospitals. Perhaps, the differences in the availability of infrastructural facilities (***Table 1***) and proportions of personnel (***Table 3***) may partly explain why higher percentage of procedures were skewed towards mission and private hospitals compared to public hospitals. Indeed, in 2018, one of the mission hospitals recorded 540 procedures per year while a public hospital reported 26 procedures per year. This gap was partly due to dearth of surgical equipment, perennial shortage of essential supplies, and limited workforce observed in the affected public hospitals and in some of the private facilities.

In the index study, the types of the surgical procedures showed a remarkable pattern. Importantly, there was widespread availability of most general surgical procedures in mission and private hospitals while none of the public hospitals performed procedures like thyroidectomy, mastectomy, chest tube insertion, fistulectomy and tracheostomy during the 5 years evaluated. A key observation in most of the health facilities was a lack of clear-cut management guidelines for anesthesia and surgery care. For instance, over 90% of the facilities have no provision for histologic assessment of appendiceal specimens, no application of mesh to repair large, recurrent and bilateral hernias and no use of a definitive airway for anesthetic management of surgical conditions with full stomach.

Generally, poor infrastructural facilities, unpredictable essential surgical supplies, limited workforce and patients’ health-seeking behavior were the key factors that drive service delivery in various facilities. Reports from previous studies in Nigeria and other parts of Africa overlapped with our findings [2,5,12,14,17,20,25].

However, investigators working in Iran found that full time specialists in surgery, anesthesia and obstetrics/gynecology were available in all the district hospitals and that basic and life-saving procedures were performed in the majority of the hospitals [26]. The above findings in the Middle East indicate that the provision of essential and emergency surgical care is feasible in district centres in emerging economies and emphasized the value of good health management plans and adequate health budgeting.

In recent times, the ‘charitable platforms in global surgery’ scheme has emerged as a useful option to upscale service delivery in LMICs, but the program is bedeviled with many draw backs [26]. In the platforms under short term surgical mission trips, reliance on local infrastructure and equipment has hampered its overall effectiveness [26]. The self- contained temporary platform mission trips and specialized hospital platforms provide more robust, effective and cost-effective care, but limited by low coverage in the LMICs [27].

The referral system noted in this study was pathetic. Most referrals from primary to secondary centres were late. Many patients were self-referred and accompanied with no referral notes. Incomplete, poorly documented referral letters were observed in many district health facilities. Reports from Tanzania, Sierra Leon and Gambia support these observations [5,12,14,20].

## Information Management and Financing

Our data showed that documentation in the clinics, wards and theatres in the majority of the facilities were incomplete and poorly organized. This has a negative effect on retrospective research works performed in the facilities. Unfortunately, due to lack of designated health record units, computer and internet access in many of the facilities, up-to-date medical information cannot be guaranteed unless a prospectively designed research project is undertaken by a dedicated research team. These findings conform with data documented in previous similar studies [2,14,17,19].

The evaluation of the financial estimates for surgical admissions was difficult, as pointed out in the result section. Notwithstanding the complex and inconsistent billing of surgical treatments, available indicators and unpublished information from interviews showed that many of the surgical patients experienced severe financial limitation before and after medical bill settlement. The low NHIS coverage and low per capita income of the patients and their families were the reasons adduced for the above observations. Other reports from Africa support this findings [14,19,20].

## Suggestions on the Strategic Plans for Improvement of Surgical Capacity in Rural Southeast Nigeria

Notwithstanding the fact that we observed significant deficits in all five main domains recognized by NSOAPs, some key strategic plans to form and sustain improvements in our socially and financially needy environment/health system have been identified.

First, improvements on infrastructure, workforce, service delivery and financing can be re-explored through revitalization of satellite health facilities (affiliated to tertiary hospitals) located at various remote communities in Southeast Nigeria. It is noteworthy that each of the five states in the southeast geopolitical zone of Nigeria has at least one federal tertiary hospital and that these tertiary health institutions have satellite centres in the district/rural areas. Unfortunately, most of the satellite facilities have low workforce, poor funding and are predominantly ill-equipped, while the few with adequate equipment to support safe surgical and anesthetic services at the district level either lack trained personnel or the equipment is run down and abandoned or dilapidated, or has been diverted or vandalized. Given the potential value of these neglected centres, urgent revitalization of the satellite centres and adoption of health policies by the federal and southeast regional governments of Nigeria to encourage urban-rural drift of trained surgical and anesthetic personnel are salutary.

Second, the state governments of southeast Nigeria, through their ministry of health, have from time to time strived to achieve a functional Public-Private Partnership (PPP) program in the health system, but regrettably, no significant progress has been recorded. Indeed, all the specialist surgical and anesthetic personnel encountered at the government-owned facilities involved in this study were based on PPP arrangement. Despite the keen disposition of the specialist surgical personnel to deliver safe surgical and anesthetic services at the district level, poor remuneration and dearth of equipment have continued to hamper the growth of this project in our environment. Nevertheless, there is a window of opportunity to harness the full potential of this program and strengthen workforce and service deliver if a robust PPP program is sought and established.

Third, improvement on information management can be triggered through a relatively convenient and fast approach especially for the mission and government-owned hospitals that have training affiliations with some of the schools of health technology where health information/record officers are trained. Curiously, southeast Nigeria is replete with both public and private-owned schools of health technology, yet most of the district centres lack trained health information personnel. The reasons for the apparent shortage of these personnel include lack of employment by hospitals, wrong posting/duty prescription by health leaders and the predilection of these health information personnel to urban/central hospitals. Unavailability of computers and internet services at most district health centres has been stressed, but in the event this challenge is overcome through special intervention by government, private sectors or PPP, health information management in rural southeast Nigeria will receive a significant boost.

Fourth, the difficulty posed by inadequate financing of health systems and low per capita income of patients in southeast Nigeria is a tall challenge, but can be improved through a robust PPP, increase in budgetary allocation on health by the government and wider coverage of NHIS in rural southeast Nigeria. The population of people under NHIS is so low that the concept is either rudimentary or non-existent in most district hospitals in rural southeast Nigeria. In recent time, a public-private-based NHIS arrangement has emerged in Nigeria, where private health leaders under partnership with government health agencies provide “health insurance services” to the rural and semi-urban dwellers at affordable and flexible terms. However, this novel program is significantly limited by the low coverage rates and insufficient awareness of its existence by the rural south easterners. Nevertheless, if properly harnessed, it promises to provide a springboard to launch safe surgical and anesthetic services in rural southeast Nigeria.

Fifth, formulation and enforcement of health laws to forestall irresponsible practices and ensure a minimum of standard in the health facilities cannot be overemphasized. This way, morbidity and mortality rates of surgical patients can be reduced. Interestingly, most of the health laws are already in place, but strict enforcement has been hampered by poor communication and deplorable road networks limiting transfer of information and law enforcement. Moreover, job apathy by poorly remunerated law enforcement agents who often receive inducements from the defaulters and non-health workers perpetrating these havocs is worrisome. Review of the incentives of the law enforcement agents upwards is warranted.

In summary, a thorough review of the health policy system in our environment, backed by adequate legislation, is needed. Persistent advocacies by health managers and policy makers, coupled with interventions in the area of training and retraining of surgical personnel, donations by private and non-governmental agencies and enactment of relevant laws/enforcement of existing laws, are imperative in order to improve and sustain efficient surgical capacity in rural southeast Nigeria.

## Limitations

The number of facilities surveyed was relatively small and may not represent the overall picture of surgical and anesthesia capacity in southeast Nigeria. The WHO situational analysis tool used in this study, though the latest of its form, may be subject to limited reliability especially in the sections covering service delivery and workforce. This is because some authors in Ghana and Sierra Leone have called for the revision of the WHO tool which gave birth to the current version [11,12]. Moreover, some of the facilities might have improved in some of the domains assessed since the time this survey was done.

## Conclusion

Overall, there were deficits in all five main domains of NSOAPs assessed in this study. Poor infrastructure, limited workforce, inefficient information management and poor health financing were the main barriers against health delivery and growth in the district hospitals surveyed. This study is the first to assess surgical capacity in district hospital in Southeast Nigeria using the revised WHO situational analysis tool. This study provided results that will be useful to health managers, clinicians, missionaries, private health entrepreneurs and the government in planning, advocacies and interventions, as well as in surveillance of existing programs.
